# The genome sequence of the Warted Knot-Horn moth, *Acrobasis repandana *Fabricius, 1798

**DOI:** 10.12688/wellcomeopenres.23665.2

**Published:** 2026-09-29

**Authors:** Douglas Boyes, Inez Januszczak, University of Oxford and Wytham Woods Genome Acquisition Lab, Natural History Museum Genome Acquisition Lab, Darwin Tree of Life Barcoding collective, Wellcome Sanger Institute Tree of Life Management, Samples and Laboratory team, Wellcome Sanger Institute Scientific Operations: Sequencing Operations, Wellcome Sanger Institute Tree of Life Core Informatics team, Tree of Life Core Informatics collective, Darwin Tree of Life Consortium, Ryan Mitchell

**Affiliations:** 1 UK Centre for Ecology & Hydrology, Wallingford, England, UK; 2 Natural History Museum, London, England, UK; 3 Independent researcher, Sligo, County Sligo, Ireland

**Keywords:** Acrobasis repandana, Warted Knot-Horn moth, genome sequence, chromosomal, Lepidoptera

## Abstract

We present a genome assembly from an individual female specimen of *Acrobasis repandana* (Warted Knot-Horn moth; Arthropoda; Insecta; Lepidoptera; Pyralidae). The genome sequence has a total length of 620.40 megabases. Most of the assembly (99.78%) is scaffolded into 32 chromosomal pseudomolecules, including the Z and W sex chromosomes. The mitochondrial genome has also been assembled and is 15.21 kilobases in length. Gene annotation of this assembly on Ensembl identified 11,522 protein-coding genes.

## Species taxonomy

Eukaryota; Opisthokonta; Metazoa; Eumetazoa; Bilateria; Protostomia; Ecdysozoa; Panarthropoda; Arthropoda; Mandibulata; Pancrustacea; Hexapoda; Insecta; Dicondylia; Pterygota; Neoptera; Endopterygota; Amphiesmenoptera; Lepidoptera; Glossata; Neolepidoptera; Heteroneura; Ditrysia; Obtectomera; Pyraloidea; Pyralidae; Phycitinae; *Acrobasis*; *Acrobasis repandana* Fabricius, 1798 (NCBI: txid1100902).

## Background

We present a chromosome-level genome sequence for *Acrobasis repandana* ( Figure 1), based on a female specimen from Wytham Woods, Oxfordshire, United Kingdom. It was sequenced as part of the Darwin Tree of Life Project, a collaborative effort to sequence all named eukaryotic species in the Atlantic Archipelago of Britain and Ireland ( Blaxter *et al.*, 2022).

**Figure 1.**
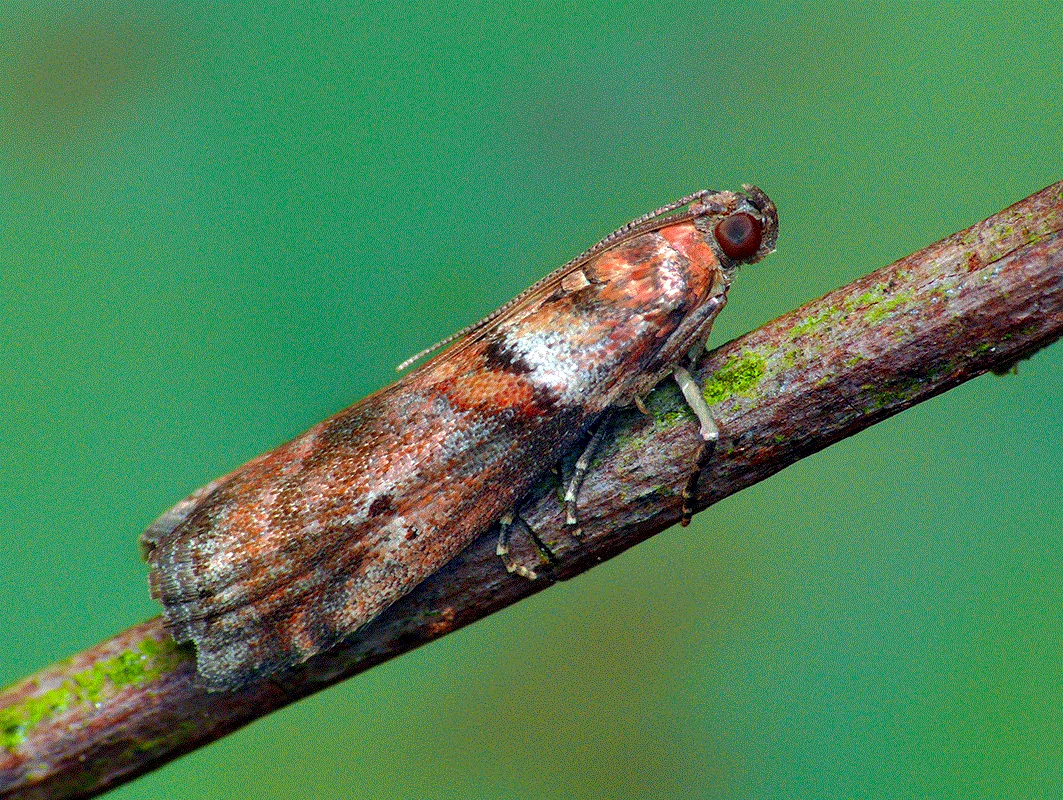
Photograph of *Acrobasis repandana* by Patrick Clement via Flickr. Not the specimen used for genome sequencing.

## Genome sequence report

The genome of *Acrobasis repandana* was sequenced using Pacific Biosciences single-molecule HiFi long reads, generating a total of 43.03 Gb (gigabases) from 3.93 million reads, providing an estimated 36-fold coverage. Primary assembly contigs were scaffolded with chromosome conformation Hi-C data, which produced 108.14 Gb from 716.19 million reads. Specimen and sequencing details are summarised in Table 1.

**Table 1.** Specimen and sequencing data for *Acrobasis repandana.*

**Project information**			
**Study title**	Acrobasis repandana (warted knot-horn)		
**Umbrella BioProject**	PRJEB65199		
**Species**	*Acrobasis repandana*		
**BioSample**	SAMEA7701484		
**NCBI taxonomy ID**	1100902		
**Specimen information**			
**Technology**	**ToLID**	**BioSample accession**	** Organism part**
**PacBio long read sequencing**	ilAcrRepa1	SAMEA7701659	Whole organism
**Hi-C sequencing**	ilAcrRepa3	SAMEA112963129	Whole organism
**Sequencing information**			
**Platform**	**Run accession**	**Read count**	**Base count (Gb)**
**Hi-C Illumina NovaSeq 6000**	ERR11872558	7.16e+08	108.14
**PacBio Sequel IIe**	ERR11867202	2.04e+06	19.88
**PacBio Sequel IIe**	ERR11867200	6.23e+05	8.08
**PacBio Sequel IIe**	ERR11867201	1.26e+06	15.06

Assembly errors were corrected by manual curation, including 26 missing joins or mis-joins and three haplotypic duplications. This reduced the scaffold number by 15.38%. The final assembly has a total length of 620.40 Mb in 54 sequence scaffolds, with 23 gaps, and a scaffold N50 of 22.2 Mb ( Table 2).

**Table 2.** Genome assembly data for *Acrobasis repandana*, ilAcrRepa1.1.

**Genome assembly**		
Assembly name	ilAcrRepa1.1	
Assembly accession	GCA_963576875.1	
*Accession of alternate haplotype*	*GCA_963576835.1*	
Span (Mb)	620.40	
Number of contigs	78	
Number of scaffolds	54	
Longest scaffold (Mb)	29.01	
Assembly metrics *		***Benchmark***
Contig N50 length (Mb)	20.9	≥ *1 Mb*
Scaffold N50 length (Mb)	22.2	*= chromosome N50*
Consensus quality (QV)	64.0	≥ *40*
*k*-mer completeness	99.06% (combined primary and alternate haplotypes)	≥ *90%*
BUSCO v5.4.3 lineage: lepidoptera_odb10	C:99.0%[S:98.5%,D:0.5%], F:0.2%,M:0.8%,n:5,286	*S > 90%, D < 5%*
Percentage of assembly mapped to chromosomes	99.78%	≥ *90%*
Sex chromosomes	ZW	*localised homologous pairs*
Organelles	Mitochondrial genome: 15.21 kb	*complete single alleles*
**Genome annotation of assembly GCA_963576875.1 at Ensembl**		
Number of protein-coding genes	11,522	
Number of non-coding genes	1,600	
Number of gene transcripts	20,378	

* Assembly metric benchmarks are adapted from Rhie *et al.* (2021) and the Earth BioGenome Project Report on Assembly Standards September 2024.

** BUSCO scores based on the lepidoptera_odb10 BUSCO set using version 5.4.3. C = complete [S = single copy, D = duplicated], F = fragmented, M = missing, n = number of orthologues in comparison. A full set of BUSCO scores is available at https://blobtoolkit.genomehubs.org/view/Acrobasis_repandana/dataset/GCA_963576875.1/busco.

The snail plot in Figure 2 provides a summary of the assembly statistics, indicating the distribution of scaffold lengths and other assembly metrics. Figure 3 shows the distribution of scaffolds by GC proportion and coverage. Figure 4 presents a cumulative assembly plot, with separate curves representing different scaffold subsets assigned to various phyla, illustrating the completeness of the assembly.

**Figure 2.**
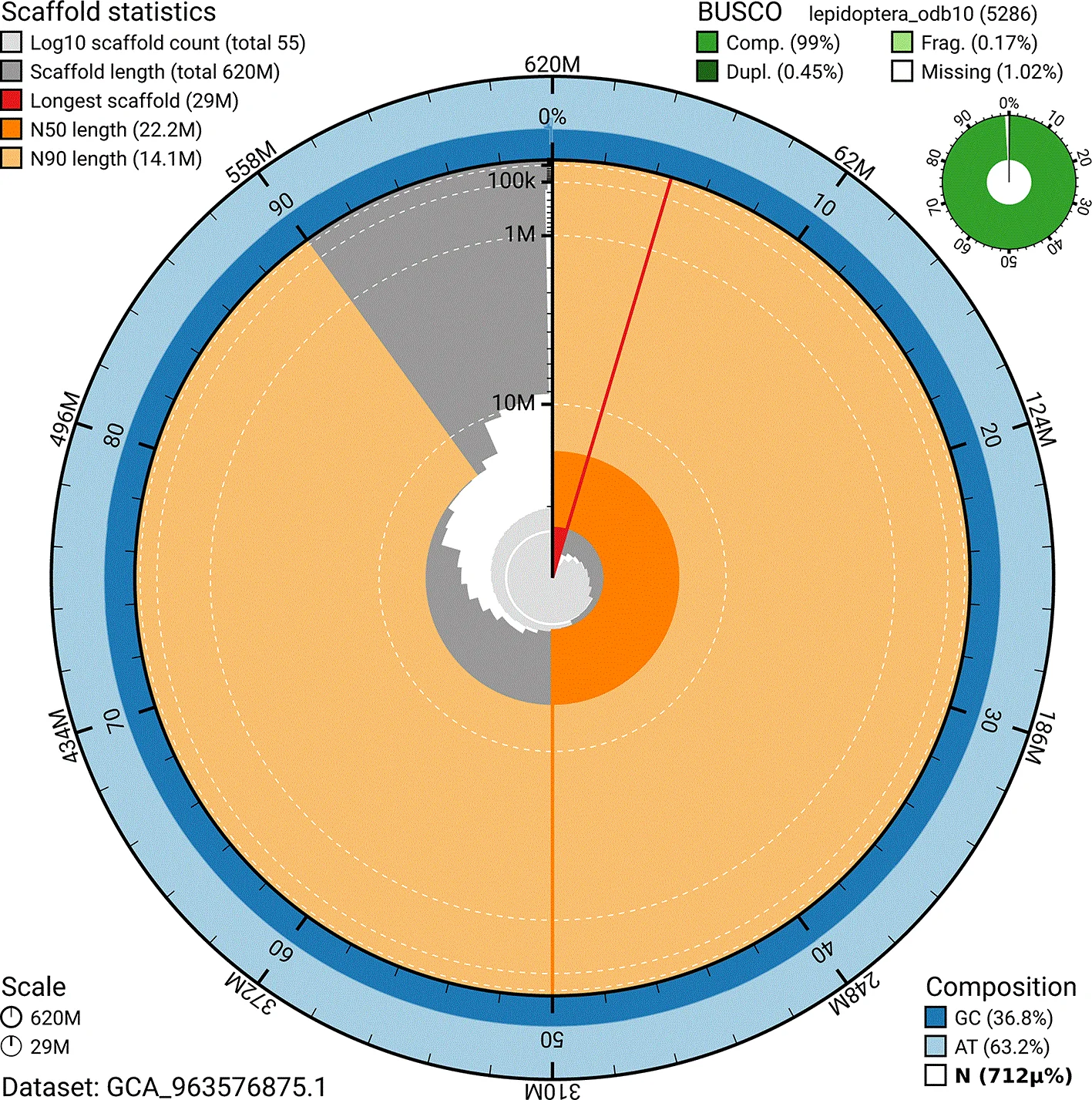
Genome assembly of *Acrobasis repandana*, ilAcrRepa1.1: metrics. The BlobToolKit snail plot provides an overview of assembly metrics and BUSCO gene completeness. The circumference represents the length of the whole genome sequence, and the main plot is divided into 1,000 bins around the circumference. The outermost blue tracks display the distribution of GC, AT, and N percentages across the bins. Scaffolds are arranged clockwise from longest to shortest and are depicted in dark grey. The longest scaffold is indicated by the red arc, and the deeper orange and pale orange arcs represent the N50 and N90 lengths. A light grey spiral at the centre shows the cumulative scaffold count on a logarithmic scale. A summary of complete, fragmented, duplicated, and missing BUSCO genes in the lepidoptera_odb10 set is presented at the top right. An interactive version of this figure is available at https://blobtoolkit.genomehubs.org/view/GCA_963576875.1/dataset/GCA_963576875.1/snail.

**Figure 3.**
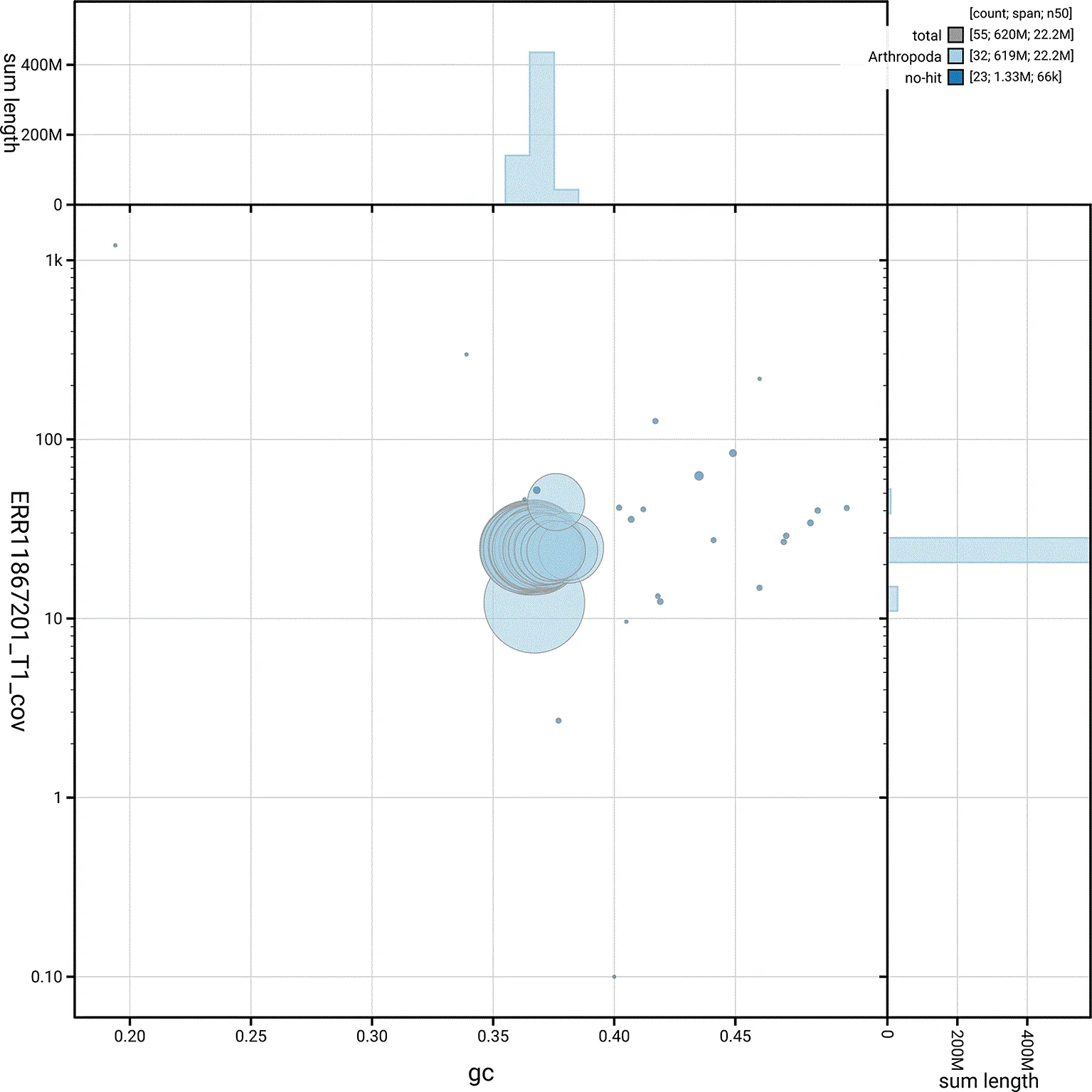
Genome assembly of *Acrobasis repandana*, ilAcrRepa1.1: BlobToolKit GC-coverage plot showing sequence coverage (vertical axis) and GC content (horizontal axis). The circles represent scaffolds, with the size proportional to scaffold length and the colour representing phylum membership. The histograms along the axes display the total length of sequences distributed across different levels of coverage and GC content. An interactive version of this figure is available at https://blobtoolkit.genomehubs.org/view/GCA_963576875.1/dataset/GCA_963576875.1/blob.

**Figure 4.**
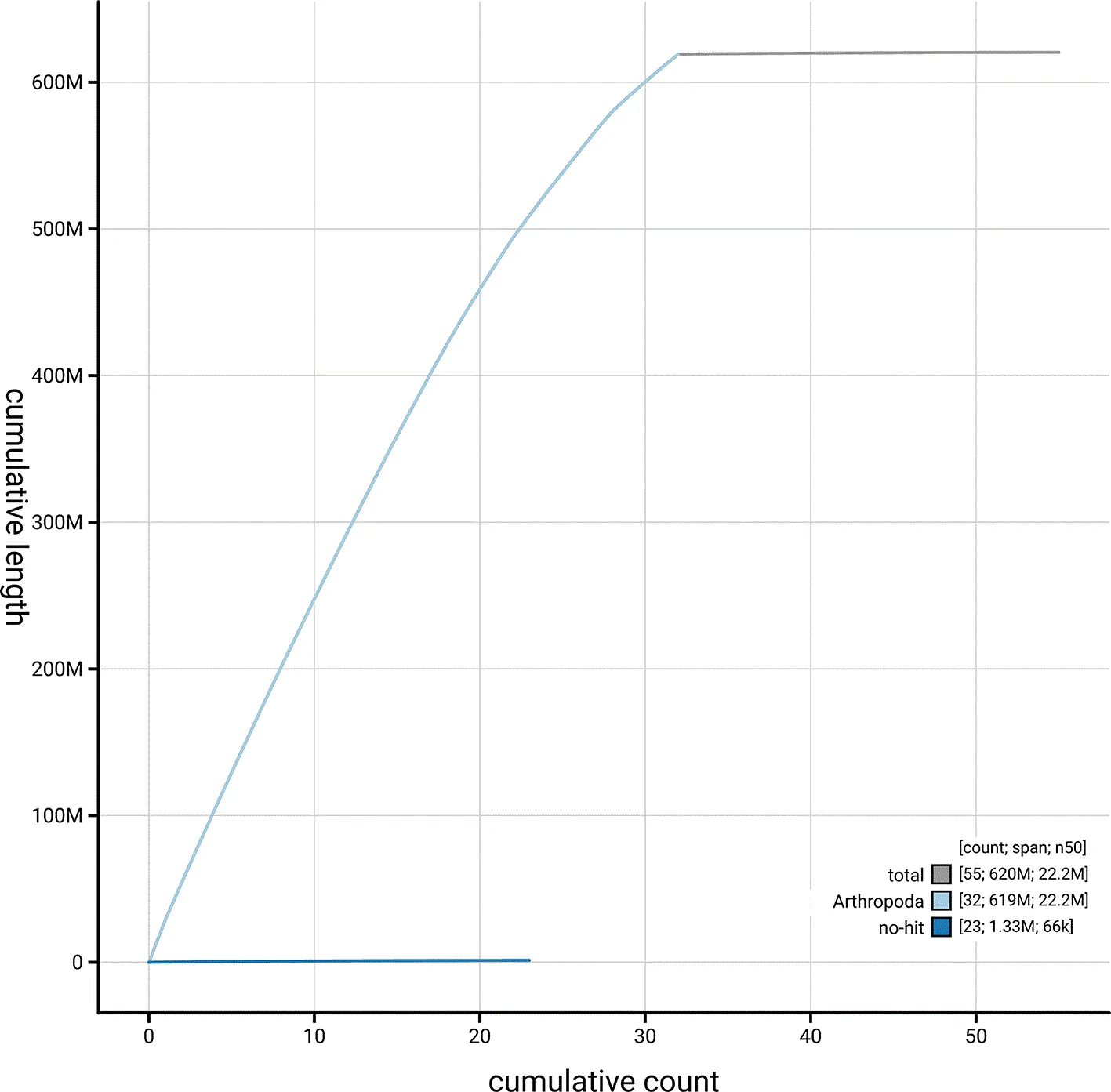
Genome assembly of *Acrobasis repandana* ilAcrRepa1.1: BlobToolKit cumulative sequence plot. The grey line shows cumulative length for all scaffolds. Coloured lines show cumulative lengths of scaffolds assigned to each phylum using the buscogenes taxrule. An interactive version of this figure is available at https://blobtoolkit.genomehubs.org/view/GCA_963576875.1/dataset/GCA_963576875.1/cumulative.

Most of the assembly sequence (99.78%) was assigned to 32 chromosomal-level scaffolds, representing 30 autosomes and the Z and W sex chromosomes. These chromosome-level scaffolds, confirmed by the Hi-C data, are named in order of size ( Figure 5; Table 3). During manual curation, chromosomes W and Z were assigned by read coverage statistics.

**Figure 5.**
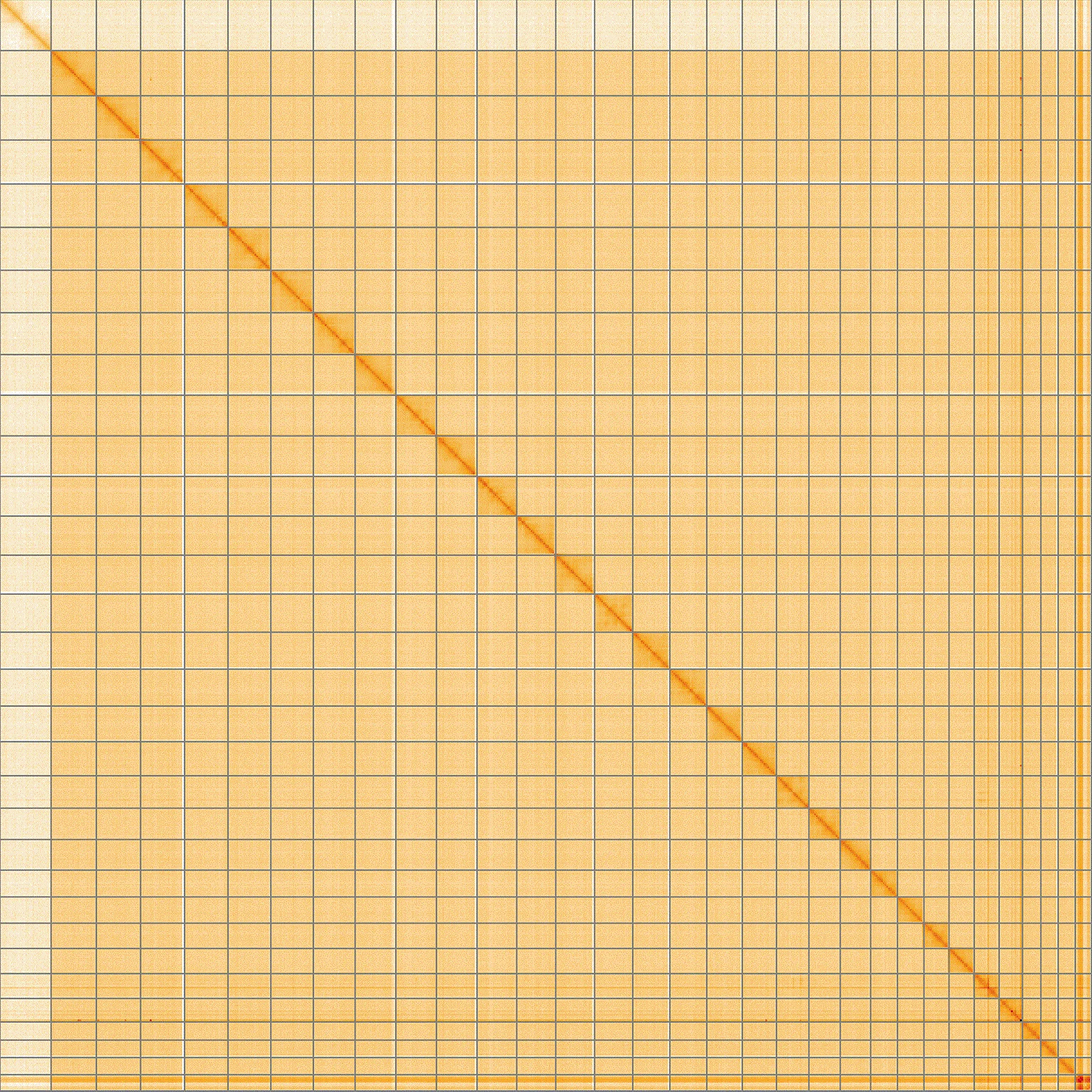
Genome assembly of *Acrobasis repandana* ilAcrRepa1.1: Hi-C contact map of the ilAcrRepa1.1 assembly, visualised using HiGlass. Chromosomes are shown in order of size from left to right and top to bottom. An interactive version of this figure may be viewed at https://genome-note-higlass.tol.sanger.ac.uk/l/?d=CnJqXb37SUC-ktxRbhhHxQ .

**Table 3.** Chromosomal pseudomolecules in the genome assembly of *Acrobasis repandana*, ilAcrRepa1.

INSDC accession	Name	Length (Mb)	GC%
OY756215.1	1	25.66	36.5
OY756216.1	2	25.16	36.5
OY756217.1	3	24.87	37.0
OY756218.1	4	24.61	36.5
OY756219.1	5	24.31	36.5
OY756220.1	6	24.1	36.5
OY756221.1	7	23.7	36.5
OY756222.1	8	23.11	36.5
OY756223.1	9	23.06	36.5
OY756224.1	10	23.01	37.0
OY756225.1	11	22.47	36.5
OY756226.1	12	22.18	36.5
OY756227.1	13	22.1	36.5
OY756228.1	14	21.64	36.5
OY756229.1	15	20.97	36.5
OY756230.1	16	20.95	36.5
OY756231.1	17	20.19	37.0
OY756232.1	18	19.35	37.0
OY756233.1	19	18.39	37.0
OY756234.1	20	17.77	36.5
OY756235.1	21	17.29	37.0
OY756236.1	22	15.23	37.0
OY756237.1	23	14.98	37.0
OY756238.1	24	14.28	37.5
OY756239.1	25	14.23	37.0
OY756240.1	26	14.13	38.0
OY756241.1	27	13.28	37.5
OY756242.1	28	10.37	37.5
OY756243.1	29	9.85	38.0
OY756244.1	30	9.7	37.5
OY756245.1	W	9.18	37.5
OY756214.1	Z	29.01	36.5
OY756246.1	MT	0.02	20.0

While not fully phased, the assembly deposited is of one haplotype. Contigs corresponding to the second haplotype have also been deposited. The mitochondrial genome was also assembled and can be found as a contig within the multifasta file of the genome submission, and as a separate fasta file with accession OY756246.1.

The final assembly has a Quality Value (QV) of 64.0 and *k*-mer completeness of 99.06% (combined primary assembly and alternate haplotype). BUSCO (v5.4.3) analysis using the lepidoptera_odb10 reference set ( *n* = 5,286) indicated a completeness score of 99.0% (single = 98.5%, duplicated = 0.5%). The assembly achieves the EBP reference standard of 6.C.64. Other quality metrics are given in Table 2.

## Genome annotation report

The *Acrobasis repandana* genome assembly (GCA_963576875.1) was annotated at the European Bioinformatics Institute (EBI) on Ensembl Rapid Release. The resulting annotation includes 20,378 transcribed mRNAs from 11,522 protein-coding and 1,600 non-coding genes ( Table 2; https://rapid.ensembl.org/Acrobasis_repandana_GCA_963576875.1/Info/Index).Theaverage transcript length is 16,452.98, with 1.55 coding transcripts per gene and 6.98 exons per transcript.

## Methods

### Sample acquisition and DNA barcoding

An adult female *Acrobasis repandana* (specimen ID Ox000620, ToLID ilAcrRepa1) was collected from Wytham Woods, Berkshire, United Kingdom (latitude 51.77, longitude −1.34) on 2020-07-05, using a light trap. The specimen was collected and identified by Douglas Boyes (University of Oxford) and preserved on dry ice.

The specimen used for Hi-C sequencing (specimen ID NHMUK015059184, ToLID ilAcrRepa3) was collected from Marston Marsh, Norwich, England, United Kingdom (latitude 52.6, longitude 1.27) on 2022-07-04. The specimen was collected by Inez Januszczak (Natural History Museum) and identified by Ryan Mitchell (National Museums Northern Ireland) and preserved by dry freezing at −80 °C.

The initial identification was verified by an additional DNA barcoding process according to the framework developed by Twyford *et al*. (2024). A small sample was dissected from the specimens and stored in ethanol, while the remaining parts were shipped on dry ice to the Wellcome Sanger Institute (WSI). The tissue was lysed, the COI marker region was amplified by PCR, and amplicons were sequenced and compared to the BOLD database, confirming the species identification ( Crowley *et al.*, 2023). Following whole genome sequence generation, the relevant DNA barcode region was also used alongside the initial barcoding data for sample tracking at the WSI ( Twyford *et al.*, 2024). The standard operating procedures for Darwin Tree of Life barcoding have been deposited on protocols.io ( Beasley *et al.*, 2023).

### Nucleic acid extraction

The workflow for high molecular weight (HMW) DNA extraction at the Wellcome Sanger Institute (WSI) Tree of Life Core Laboratory includes a sequence of procedures: sample preparation and homogenisation, DNA extraction, fragmentation and purification. Detailed protocols are available on protocols.io ( Denton *et al.*, 2023b).

The ilAcrRepa1 sample was prepared for DNA extraction by weighing and dissecting it on dry ice ( Jay *et al.*, 2023). Tissue from the whole organism was homogenised using a PowerMasher II tissue disruptor ( Denton *et al.*, 2023a). HMW DNA was extracted using the Automated MagAttract v1 protocol ( Sheerin *et al.*, 2023). DNA was sheared into an average fragment size of 12–20 kb in a Megaruptor 3 system ( Todorovic *et al.*, 2023). Sheared DNA was purified by solid-phase reversible immobilisation, using AMPure PB beads to eliminate shorter fragments and concentrate the DNA ( Strickland *et al.*, 2023). The concentration of the sheared and purified DNA was assessed using a Nanodrop spectrophotometer and a Qubit Fluorometer using the Qubit dsDNA High Sensitivity Assay kit. The fragment size distribution was evaluated by running the sample on the FemtoPulse system.

### Hi-C preparation

Whole organism tissue of the ilAcrRepa3 sample was processed at the WSI Scientific Operations core, using the Arima-HiC v2 kit. Tissue (stored at −80 °C) was fixed, and the DNA crosslinked using a TC buffer with 22% formaldehyde. After crosslinking, the tissue was homogenised using the Diagnocine Power Masher-II and BioMasher-II tubes and pestles. Following the kit manufacturer’s instructions, crosslinked DNA was digested using a restriction enzyme master mix. The 5′-overhangs were then filled in and labelled with biotinylated nucleotides and proximally ligated. An overnight incubation was carried out for enzymes to digest remaining proteins and for crosslinks to reverse. A clean up was performed with SPRIselect beads prior to library preparation.

### Library preparation and sequencing

For PacBio DNA sequencing, samples were required to have an average fragment size exceeding 8 kb and a total mass over 400 ng to proceed to the low input SMRTbell Prep Kit 3.0 protocol (Pacific Biosciences, California, USA) depending on genome size and sequencing depth required. The kit includes the reagents required for end repair/A-tailing, adapter ligation, post-ligation SMRTbell bead cleanup, and nuclease treatment. Following the manufacturer’s instructions, size selection and clean up was carried out using diluted AMPure PB beads (Pacific Biosciences). DNA concentration was quantified using the Qubit Fluorometer v4.0 (Thermo Fisher Scientific) with Qubit 1X dsDNA HS assay kit and the final library fragment size analysis was carried out using the Agilent Femto Pulse Automated Pulsed Field CE Instrument (Agilent Technologies) and gDNA 55 kb BAC analysis kit. Samples were sequenced using the Sequel IIe system (Pacific Biosciences, California, USA). The concentration of the library loaded onto the Sequel IIe was in the range of 40–135 pM. The SMRT link software, a PacBio web-based end-to-end workflow manager, was used to set-up and monitor the run, as well as perform primary and secondary analysis of the data upon completion.

For Hi-C library preparation, DNA was fragmented to a size of 400 to 600 bp using a Covaris E220 sonicator. The DNA was then enriched, barcoded, and amplified using the NEBNext Ultra II DNA Library Prep Kit following manufacturers’ instructions. The Hi-C sequencing was performed using paired-end sequencing with a read length of 150 bp on an Illumina NovaSeq 6000 instrument.

### Genome assembly, curation and evaluation

***Assembly***

The HiFi reads were first assembled using Hifiasm ( Cheng *et al.*, 2021) with the --primary option. Haplotypic duplications were identified and removed using purge_dups ( Guan *et al.*, 2020). The Hi-C reads were mapped to the primary contigs using bwa-mem2 ( Vasimuddin *et al.*, 2019). The contigs were further scaffolded using the provided Hi-C data ( Rao *et al.*, 2014) in YaHS ( Zhou *et al.*, 2023) using the --break option for handling potential misassemblies. The scaffolded assemblies were evaluated using Gfastats ( Formenti *et al.*, 2022), BUSCO ( Manni *et al.*, 2021) and MERQURY.FK ( Rhie *et al.*, 2020).

The mitochondrial genome was assembled using MitoHiFi ( Uliano-Silva *et al.*, 2023), which runs MitoFinder ( Allio *et al.*, 2020) and uses these annotations to select the final mitochondrial contig and to ensure the general quality of the sequence.

***Assembly curation***

The assembly was decontaminated using the Assembly Screen for Cobionts and Contaminants (ASCC) pipeline (article in preparation). Flat files and maps used in curation were generated in TreeVal ( Pointon *et al.*, 2023). Manual curation was primarily conducted using PretextView ( Harry, 2022), with additional insights provided by JBrowse2 ( Diesh *et al.*, 2023) and HiGlass ( Kerpedjiev *et al.*, 2018). Scaffolds were visually inspected and corrected as described by Howe *et al*. (2021). Any identified contamination, missed joins, and mis-joins were corrected, and duplicate sequences were tagged and removed. The curation process is documented at https://github.com/sanger-tol/curation-resources .

***Assembly quality assessment***

The Merqury.FK tool ( Rhie *et al.*, 2020), run in a Singularity container ( Kurtzer *et al.*, 2017), was used to evaluate *k*-mer completeness and assembly quality for the primary and alternate haplotypes using the *k*-mer databases ( *k* = 31) that were computed prior to genome assembly. The analysis outputs included assembly QV scores and completeness statistics.

A Hi-C contact map was produced for the final version of the assembly. The Hi-C reads were aligned using bwa-mem2 ( Vasimuddin *et al.*, 2019) and the alignment files were combined using SAMtools ( Danecek *et al.*, 2021). The Hi-C alignments were converted into a contact map using BEDTools ( Quinlan & Hall, 2010) and the Cooler tool suite (Abdennur & Mirny, 2020). The contact map is visualised in HiGlass ( Kerpedjiev *et al.*, 2018).

The blobtoolkit pipeline is a Nextflow port of the previous Snakemake Blobtoolkit pipeline ( Challis *et al.*, 2020). It aligns the PacBio reads in SAMtools and minimap2 ( Li, 2018) and generates coverage tracks for regions of fixed size. In parallel, it queries the GoaT database ( Challis *et al.*, 2023) to identify all matching BUSCO lineages to run BUSCO ( Manni *et al.*, 2021). For the three domain-level BUSCO lineages, the pipeline aligns the BUSCO genes to the UniProt Reference Proteomes database ( Bateman *et al.*, 2023) with DIAMOND blastp (Buchfink *et al.*, 2021). The genome is also divided into chunks according to the density of the BUSCO genes from the closest taxonomic lineage, and each chunk is aligned to the UniProt Reference Proteomes database using DIAMOND blastx. Genome sequences without a hit are chunked using seqtk and aligned to the NT database with blastn ( Altschul *et al.*, 1990). The blobtools suite combines all these outputs into a blobdir for visualisation.

The blobtoolkit pipeline was developed using nf-core tooling ( Ewels *et al.*, 2020) and MultiQC ( Ewels *et al.*, 2016), relying on the Conda package manager, the Bioconda initiative ( Grüning *et al.*, 2018), the Biocontainers infrastructure ( da Veiga Leprevost *et al.*, 2017), as well as the Docker ( Merkel, 2014) and Singularity ( Kurtzer *et al.*, 2017) containerisation solutions.

Table 4 contains a list of relevant software tool versions and sources.

**Table 4.** Software tools: versions and sources.

Software tool	Version	Source
BEDTools	2.30.0	https://github.com/arq5x/bedtools2
BLAST	2.14.0	ftp://ftp.ncbi.nlm.nih.gov/blast/executables/blast+/
BlobToolKit	4.3.7	https://github.com/blobtoolkit/blobtoolkit
BUSCO	5.4.3 and 5.5.0	https://gitlab.com/ezlab/busco
bwa-mem2	2.2.1	https://github.com/bwa-mem2/bwa-mem2
Cooler	0.8.11	https://github.com/open2c/cooler
DIAMOND	2.1.8	https://github.com/bbuchfink/diamond
fasta_windows	0.2.4	https://github.com/tolkit/fasta_windows
FastK	427104ea91c78c3b8b8b49f1a7d6bbeaa869ba1c	https://github.com/thegenemyers/FASTK
Gfastats	1.3.6	https://github.com/vgl-hub/gfastats
GoaT CLI	0.2.5	https://github.com/genomehubs/goat-cli
Hifiasm	0.19.8-r587	https://github.com/chhylp123/hifiasm
HiGlass	44086069ee7d4d3f6f3f0012569789ec138f42b84aa44357826c0b6753eb28de	https://github.com/higlass/higlass
Merqury.FK	d00d98157618f4e8d1a9190026b19b471055b22e	https://github.com/thegenemyers/MERQURY.FK
MitoHiFi	3	https://github.com/marcelauliano/MitoHiFi
MultiQC	1.14, 1.17, and 1.18	https://github.com/MultiQC/MultiQC
NCBI Datasets	15.12.0	https://github.com/ncbi/datasets
Nextflow	23.04.0–5857	https://github.com/nextflow-io/nextflow
PretextView	0.2.5	https://github.com/sanger-tol/PretextView
purge_dups	1.2.5	https://github.com/dfguan/purge_dups
samtools	1.16.1, 1.17, and 1.18	https://github.com/samtools/samtools
sanger-tol/ ascc	-	https://github.com/sanger-tol/ascc
sanger-tol/ blobtoolkit	0.6.0	https://github.com/sanger-tol/blobtoolkit
Seqtk	1.3	https://github.com/lh3/seqtk
Singularity	3.9.0	https://github.com/sylabs/singularity
TreeVal	1.0.0	https://github.com/sanger-tol/treeval
YaHS	1.2a.2	https://github.com/c-zhou/yahs

### Genome annotation

The Ensembl Genebuild annotation system ( Aken *et al.*, 2016) was used to generate annotation for the *Acrobasis repandana* assembly (GCA_963576875.1) in Ensembl Rapid Release at the EBI. Annotation was created primarily through alignment of transcriptomic data to the genome, with gap filling via protein-to-genome alignments of a select set of proteins fromUniProt ( UniProt Consortium, 2019).

### Wellcome Sanger Institute – Legal and Governance

The materials that have contributed to this genome note have been supplied by a Darwin Tree of Life Partner. The submission of materials by a Darwin Tree of Life Partner is subject to the **‘Darwin Tree of Life Project Sampling Code of Practice’**, which can be found in full on the Darwin Tree of Life website here. By agreeing with and signing up to the Sampling Code of Practice, the Darwin Tree of Life Partner agrees they will meet the legal and ethical requirements and standards set out within this document in respect of all samples acquired for, and supplied to, the Darwin Tree of Life Project.

Further, the Wellcome Sanger Institute employs a process whereby due diligence is carried out proportionate to the nature of the materials themselves, and the circumstances under which they have been/are to be collected and provided for use. The purpose of this is to address and mitigate any potential legal and/or ethical implications of receipt and use of the materials as part of the research project, and to ensure that in doing so we align with best practice wherever possible. The overarching areas of consideration are:

• Ethical review of provenance and sourcing of the material
• Legality of collection, transfer and use (national and international)

Each transfer of samples is further undertaken according to a Research Collaboration Agreement or Material Transfer Agreement entered into by the Darwin Tree of Life Partner, Genome Research Limited (operating as the Wellcome Sanger Institute), and in some circumstances other Darwin Tree of Life collaborators.

## Author information

Members of the University of Oxford and Wytham Woods Genome Acquisition Lab are listed here: https://doi.org/10.5281/zenodo.12157525.

Members of the Natural History Museum Genome Acquisition Lab are listed here: https://doi.org/10.5281/zenodo.12159242.

Members of the Darwin Tree of Life Barcoding collective are listed here: https://doi.org/10.5281/zenodo.12158331.

Members of the Wellcome Sanger Institute Tree of Life Management, Samples and Laboratory team are listed here: https://doi.org/10.5281/zenodo.12162482.

Members of Wellcome Sanger Institute Scientific Operations: Sequencing Operations are listed here: https://doi.org/10.5281/zenodo.12165051.

Members of the Wellcome Sanger Institute Tree of Life Core Informatics team are listed here: https://doi.org/10.5281/zenodo.12160324.

Members of the Tree of Life Core Informatics collective are listed here: https://doi.org/10.5281/zenodo.12205391.

Members of the Darwin Tree of Life Consortium are listed here: https://doi.org/10.5281/zenodo.4783558.

## Data Availability

European Nucleotide Archive: Acrobasis repandana (warted knot-horn). Accession number PRJEB65199; https://identifiers.org/ena.embl/PRJEB65199. The genome sequence is released openly for reuse. The *Acrobasis repandana* genome sequencing initiative is part of the Darwin Tree of Life (DToL) project. All raw sequence data and the assembly have been deposited in INSDC databases. Raw data and assembly accession identifiers are reported in Table 1 and Table 2.
