# Supplementary material for: The genome sequence of the Warted Knot-Horn moth, Acrobasis repandana Fabricius, 1798
Source: Wellcome Open Res. 2026 Sep 29;10:50. Originally published 2025 Feb 7. [Version 2] doi: 10.12688/wellcomeopenres.23665.2 (PMC12004066; doi:10.12688/wellcomeopenres.23665.2)
Supplement: Peer review history

# Reviewer report: Reviewer response for version 1

**DOI:** 10.21956/wellcomeopenres.26107.r120849

**Published:** 2025-04-16

**Authors:** Savarimuthu Ignacimuthu, Sivasankaran Kuppusamy

**Affiliations:** 1 St Xavier's College, Palayamkottai, Tamil Nadu, India; 2 Entomology Research Institute, Loyola College, Chennai, Tamil Nadu, India

The authors have done chromosome-level genome assembly of Warted Knot-Horn moth, *Acrobasis repandana *Fabricius, 1798. They assembled the sequence using appropriate softwares and obtained the genome 620.40 megabases in size. The assembly was scaffolded into 32 chromosomes. The materials and methods were properly described. 

**Minor comments on the manuscript**

 The first sentence of the background may be modified as “We present a chromosome-level genome assembly for *Acrobasis repandana *Fabricius, 1798…”

 The authors consistently used the complete genus name, *Acrobasis repandana*, throughout the manuscript. If possible, to use the full genus name at first, and later to shorten it to *A. repandana.*

 The complete length of the mitochondrial genome sequence was not given in the text. The length of the sequence can be included in the text. 

 The manuscript is well prepared therefore I confirm that the manuscript meets the necessary scientific standard and is suitable for indexing.

Are sufficient details of methods and materials provided to allow replication by others?

Yes

Is the rationale for creating the dataset(s) clearly described?

Yes

Are the datasets clearly presented in a useable and accessible format?

Yes

Are the protocols appropriate and is the work technically sound?

Yes

Reviewer Expertise:

Molecular biology

We confirm that we have read this submission and believe that we have an appropriate level of expertise to confirm that it is of an acceptable scientific standard.

# Reviewer report: Reviewer response for version 1

**DOI:** 10.21956/wellcomeopenres.26107.r118569

**Published:** 2025-02-24

**Authors:** Jinfeng Chen

**Affiliations:** 1 Institute of Crop Sciences, State Key Laboratory of Integrated Management of Pest Insects and Rodents, Institute of Zoology, Chinese Academy of Agricultural Sciences, Beijing, China

The manuscript describes the genome assembly of the Warted Knot-Horn moth, *Acrobasis repandana*. The authors generated 36-fold genome coverage HiFi reads and assembled a 620.40 of very high quality as shown by the 99% BUSCO score and many other parameters.  The methods were well described and the related data were available in the public database. Therefore, I think this is a well-organized manuscript for describing a genome. I don't any further questions on indexing it.

Are sufficient details of methods and materials provided to allow replication by others?

Yes

Is the rationale for creating the dataset(s) clearly described?

Yes

Are the datasets clearly presented in a useable and accessible format?

Yes

Are the protocols appropriate and is the work technically sound?

Yes

Reviewer Expertise:

Comparative genomics and transposable elements

I confirm that I have read this submission and believe that I have an appropriate level of expertise to confirm that it is of an acceptable scientific standard.
